# The Response of Lentil (*Lens culinaris* Medik.) to Soil Moisture and Heat Stress Under Different Dates of Sowing and Foliar Application of Micronutrients

**DOI:** 10.3389/fpls.2021.679469

**Published:** 2021-07-22

**Authors:** Visha Kumari Venugopalan, Rajib Nath, Kajal Sengupta, Arpita Nalia, Saon Banerjee, Malamal A. Sarath Chandran, Ulkar Ibrahimova, Eldessoky S. Dessoky, Attia O. Attia, Mohamed M. Hassan, Akbar Hossain

**Affiliations:** ^1^Department of Agronomy, Bidhan Chandra Krishi Viswavidalya, Haringhata, India; ^2^Department of Agricultural Meteorology and Physics, Bidhan Chandra Krishi Viswavidalya, Haringhata, India; ^3^Institute of Molecular Biology and Biotechnologies Azerbaijan National Academy of Sciences, Baku, Azerbaijan; ^4^Department of Biology, College of Science, Taif University, Taif, Saudi Arabia; ^5^Department of Agronomy, Bangladesh Wheat and Maize Research Institute, Dinajpur, Bangladesh

**Keywords:** lentil, moisture stress, heat stress, sowing times, SDDI, micronutrients

## Abstract

Soil moisture and air temperature stress are the two major abiotic factors limiting lentil (*Lens culinaris* Medik.) growth and productivity in the humid tropics. Field experiments were conducted during winter seasons (November to March) of 2018–2019 and 2019–2020 on clay loam soil (AericHaplaquept) of Eastern India to cultivate rainfed lentil, with residual moisture. The objective was to study the effect of different time of sowing and foliar spray of micronutrients in ameliorating the effect of heat and moisture stress lentil crop experience in its reproductive stage. The study was conducted with two different dates of sowing, November and December, as main plot treatment and micronutrients foliar spray of boron, iron, and zinc either alone or in combination as subplot treatment. No foliar spray treatment was considered as a control. The soil moisture content is depleted from 38 to 18% (sowing to harvest) during November sowing; however, in December sowing, the depletion is from 30 to 15%. The foliar spray of micronutrients helped to have a better canopy cover and thus reduced soil evaporation during the later stages of crop growth when the temperature was beyond the threshold temperature of the crop. Crop growth rate (CGR) and biomass were significantly higher (*p* ≤ 0.05) for November sown crop and with foliar spray of boron and iron (FSB + FE) micronutrients. In the later stages of the crop when the soil moisture started depleting with no precipitation, the canopy temperature increased compared with air temperature, leading to positive values of Stress Degree Days (SDD) index. Delay in sowing reduced the duration by 11.4 days (113.5 vs. 102.1 days), resulting in varied accumulated Growing Degree Days (GDD). FSB + FE resulted in the highest yield in both years (1,436 and 1,439 kg ha^−1^). The results of the study concluded that the optimum time of sowing and foliar spray of micronutrients may be helpful to alleviate the soil moisture and heat stress for the sustainability of lentil production in the subtropical region.

## Introduction

Abiotic stresses, such as high temperature and moisture, are major environmental factors that limit the growth and productivity of crops. Climate change has increased the intensity of adverse crop environment, resulting in severe economic loss in agricultural and horticultural crops (Beck et al., 2007). Elevated temperatures and moisture stress can cause various morpho-anatomical, physiological, reproductive, and biochemical changes in plants, which can affect plant growth and development and ultimately lead to reduction in economic yield (Bita and Gerats, 2013). High temperature is very often related to reducing the availability of water (Barnabás et al., 2008). The effects of abiotic stresses, mainly at the time of the reproductive stage of plants, are gaining attention, as they are a serious threat to the productivity of leguminous crops by reducing pollen viability, fertilization, and pod set (Gaur et al., 2015). The incidence of drought, accompanied by heat stress, is likely to increase in the near future (IPCC, 2014), which highlights the need to investigate a more economical management option to reduce its adverse effect.

Lentil (*Lens culinaris* L.) is the second most important cool-season legume crop in India (Ram and Punia, 2018). It covers an area of 1.51 million ha with a production of 1.56 million tons and productivity of 1,032 kg ha^−1^ (Directorate of Economics and Statistics, 2020). Lentil is generally grown as a rain-fed crop during the winter season. It can be grown on residual soil moisture without any additional irrigation in the vast fallow land in India just after the harvest of Kharif rice (previous crop). However, fluctuation in temperature limits the growth and productivity of lentil in the country. During the cool season, legumes are adapted to the low and mild temperature and, hence, show high sensitivity to heat stress, as observed in chickpea (Kaushal et al., 2013) and lentil (Sita et al., 2018). Lentil is reported for its high sensitivity to high temperature and moisture stress, particularly during the reproductive stage, leading to a drastic reduction in yield (Sita et al., 2017). It requires low temperatures at the time of vegetative growth but comparatively warm temperatures during the maturity stage: for optimum growth, the required temperature ranges from 18 to 30°C (Sinsawat et al., 2004). Temperatures above 32/20°C (max/min) at the time of flowering and pod filling in lentil can drastically reduce seed yield and quality(Delahunty et al., 2015; Bourgault et al., 2018). In addition, the cultivation of long-duration rice inhibits the sowing of lentil as a sole crop in India. Long-term trend data showed that the crop would face the adverse effect of heat and moisture stress when sown late. Apart from the temperature stress during the reproductive stage, the crop may face initial or late moisture stress because of the hard layer of puddled rice soil and depleting soil moisture as no external irrigation is provided. It has been reported that lentil is largely affected by temperature, rainfall, and sowing date (Saxena, 2009).

Foliar application of micronutrients helps in the rapid translocation when compared with soil application, which is very pertinent in mitigating stress in plants especially under late sown conditions. Exogenous application of nutrients might prove a potent tool to alleviate the deleterious impacts of heat (Waraich et al., 2012). All three micronutrients, zinc, iron, and boron, have a diverse role in plant reproductive development. Zinc is a micronutrient known for its metabolic and regulatory functions (Broadly et al., 2007). It also plays a pivotal role in the reproductive phase of the crop. Iron (Fe) is important for various biochemical pathways of plants (Briat et al., 1995; Rout and Sahoo, 2015). The impact of boron deficiency on assimilate partitioning may greatly influence the ability of plants to cope with other unfavorable environmental conditions such as soil water deficit and low supply of other nutrients. Boron plays an important role in the reproductive growth of plants (Dear and Lipsett, 1987; Dell and Huang, 1997). Various earlier literatures revealed that zinc, iron and boron can also regulate the biosynthesis of chlorophyll, improve photosynthetic rate, and, thus, alleviate the effect of stress on crops (Marschner, 2012). Thus, we hypothesize that foliar sprays of micronutrients would be effective agronomic management that can help in mitigating stress and improving yield. Against this background, the study was conducted with two objectives: (1) appropriate date of sowing and foliar spray of micronutrient will have a positive impact on CGR, biomass, LAI (leaf area index), and growth; and (2) the appropriate time of sowing and foliar spray of micronutrients will have an impact on canopy temperature, SDD index, soil water availability, and phenology of crops and better yield.

## Materials and Methods

### Site Characteristics

The field experiment was conducted during the rabi season of 2018–2019 and 2019–2020 at the Seed Farm of Bidhan Chandra Krishi Viswavidyalaya (latitude 22°58′ N and longitude 88°32′ E), Kalyani, West Bengal, India. The study site is flat and is located at an altitude of 9.75 m above mean sea level (AMSL). The soil is well-drained Gangetic alluvial soil (order: inceptisol), which belonged to the class of clayey loam with medium fertility and almost neutral in reaction. The soil was low in organic carbon (wet digestion method), available nitrogen (alkaline permanganate-oxidizable), zinc (DTPA-extractable), boron (azomethine H), and iron (DTPA extractable) (0.52%; 138 kg ha^−1^; 0.4, 0.49, and 0.45, respectively), fairly rich in available P_2_O_5_ (Bray'P), and K_2_O (NH_4_OAC-extractable) (30 and 160 kg ha^−1^, respectively).

### Treatment Description and Experimental Design

The experiment was laid in a split-plot design with three replications. The main plots were two different dates of sowing, November (normal) and December (late), and the subplots were foliar spray of different micronutrients. Treatment abbreviations along with the definition of treatments are given in Table 1. In this study, we used a popular lentil variety, namely, Moitree (“WBL 77”). It is a red, small-seeded lentil variety. This is the most preferred variety among farmers because of its medium duration and better yield. The foliar sprays were given at the flower and pod initiation stages.

**Table 1:** Treatment description and abbreviation used.

**Treatment**	**Abbreviation**
No spray (Control)	NS_C_
Foliar spray of tap water	FS_T_
Foliar spray of Zn at 0.5% (ZnSO_4_.7H_2_O)	FS_ZN_
Foliar Spray of Fe at 0.5% (FeSO_4_.7H_2_O)	FS_FE_
Foliar spray of B at 0.2% (Borax 10.5%)	FS_B_
Foliar spray of Zn at 0.5% + B at 0.2%	FS_ZN+B_
Foliar spray of Zn at 0.5% + Fe at 0.5%	FS_ZN+FE_
Foliar spray of B at 0.2% + Fe at 0.5%	FS_B+FE_
Foliar spray of Zn at 0.5% + Fe at 0.5% + B at 0.2%]	FS_ZN+FE+B_

### Crop Management

The seeds were sown at 30 cm row spacing in an experimental plot of 5 × 4 m as per the sowing time of various main plot treatments. Standard crop management practices, such as a uniform fertilizer dose of 20:40:40 kg ha^−1^ of N: P_2_O_5_ and K_2_O, and one hand weeding at 25–30 days after sowing (DAS), were given. No irrigation was provided, because lentil was grown on residual soil moisture along with little precipitation during the rabi season.

### Weather

**Figure 1:**
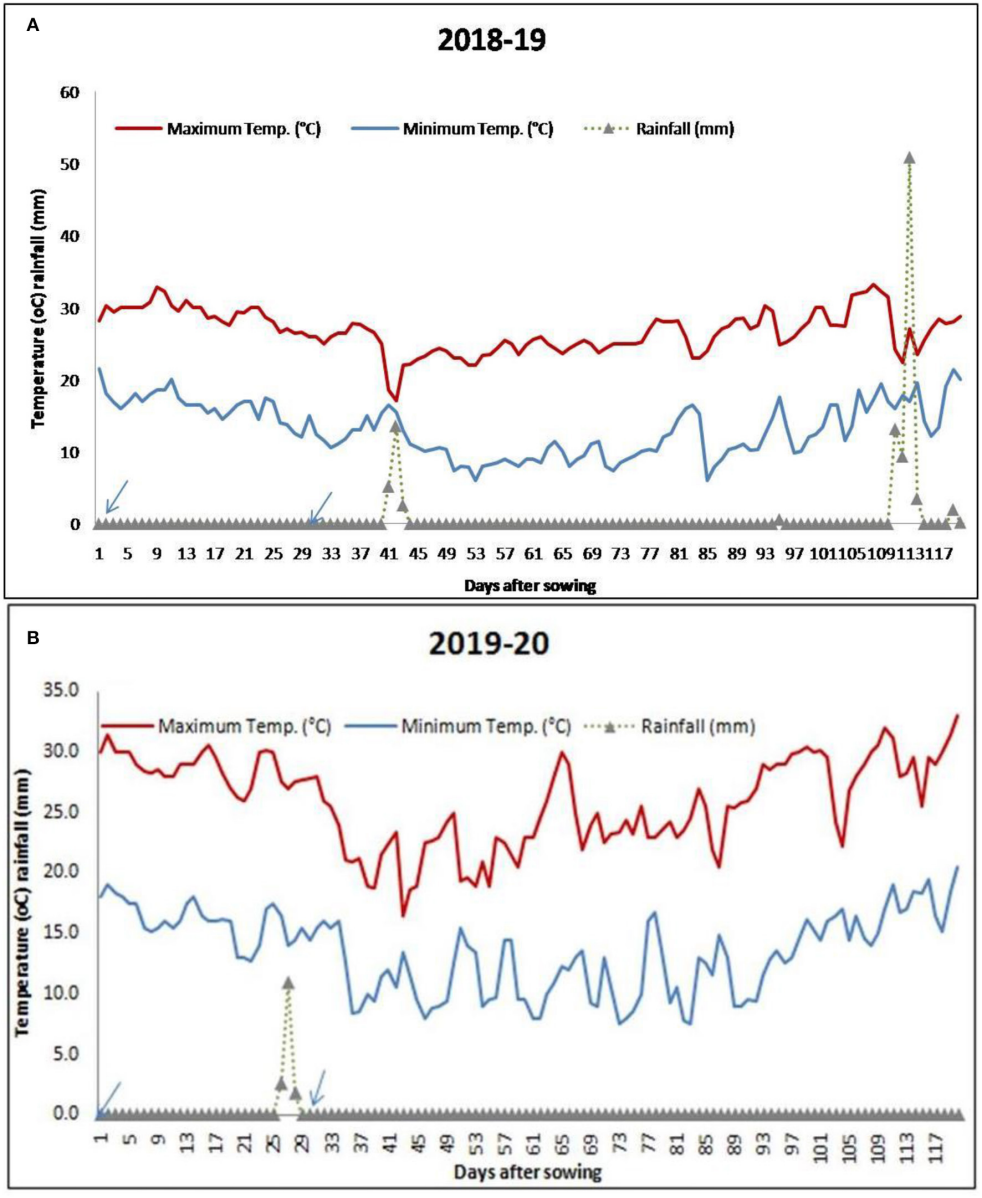
Average daily distribution of temperature and rainfall during the study period [**(A)** 2018–2019 and **(B)** 2019–2020]. Arrows indicate the sowing dates of lentil under November sowing and December sowing.

The meteorological data for the period of investigation (November 2018 to March 2019 and November 2019 to March 2020) were collected from the AICRP at the Agrometeorology unit, Directorate of Research, Kalyani, West Bengal. The mean Tmax, Tmin, and rainfall received during different growth stages are reported in Table 2 and Figure 1.

**Table 2:** Phenological-stage wise mean rainfall, and maximum and minimum temperature during the study period.

**Parameter**	**Year**	**Sowing time**	**Germination**	**Flowering**	**Pod initiation**	**Maturity**
Rainfall (mm)	2018–2019	Normal	0	21.4	0	0
		Late	5.2	13.6	0.6	51
	2019–2020	Normal	0	15.4	0	0
		Late	0	0	0	0.3
Tmax (°C)	2018–2019	Normal	29.6	26.4	24.7	25.9
		Late	25.7	24.3	26.5	29.4
	2019–2020	Normal	30.1	25.5	23.1	26.4
		Late	27	22.4	24	29.8
Tmin (°C)	2018–2019	Normal	17.7	13.6	9.2	10.7
		Late	10.5	9.6	11.9	11.5
	2019–2020	Normal	18.1	13.6	11.5	12.8
		Late	15.4	11	11.1	16.7

### Measurement of Crop Growth, Thermal Indices, Phenology, and Yield Attributes

For sampling, 20 random plants were selected from each plot, excluding border row, for taking observation on growth and yield attributes of lentil. The phenological stages (viz., emergence, flower, and pod initiation and maturity) of the crop sown on different dates were noted by a regular field inspection method. Phenophase-wise growing degree days (GDD) were calculated following Nuttonson (Nuttonson, 1955) by taking a base temperature of 5°C.

For the analysis of leaf area index (LAI) and dry biomass production of lentil, plant samples were taken from 10 randomly selected plants per plot at vegetative (30–45 DAS), flowering (45–75 DAS), pod formation (75–90 DAS), and maturity (90–105 DAS) stages. The green leaf portions were separated, and the area of the leaves was measured. Mean value per plant was used in calculating the LAI, which was derived using Equation 1:

(1) $$\begin{array}{r} \text{LAI}=\frac{Measure\;leaf\;area\;per\;plant\;\left(m^{2}\right)\times \;number\;of\;plants}{Ground\;area\;\left(m^{2}\right)} \end{array}$$

Crop growth rate (CGR) is the rate of dry biomass production per unit ground area per unit time. It was calculated using Equation 2 and expressed as g m^−2^ day^−1^.

(2) $$\begin{array}{r} CGR=\frac{W_{2}-W_{1}}{t_{2}-t_{1}}gm^{-2}day^{-1} \end{array}$$

where, W_1_ is the dry weight of the plant (g m^−2^) at time t_1_; W_2_ is the dry weight of the plant (g m^−2^) at time t_2_; (t_1_-t_2_), the time interval in days; the dry biomass was measured at the vegetative, flowering, and at pod-formation stages of the crop. The net assimilation rate (NAR g m^−2^ day^−1^) was worked out from the ratio of and LAI using Equation 3:

(3) $$\begin{array}{r} NAR=\frac{CGR}{LAI}gm^{-2}day^{-1} \end{array}$$

After lentil attained physiological maturity, grain yield was determined by hand-harvesting on the whole-plot basis from each plot, and the yield data were normalized to 14% seed moisture content.

Canopy temperature (°C) was measured at 11:30 h with the help of an infrared thermometer.

The same was used in calculating the SDD index using Equation 4:

(4) $$\begin{array}{r} SDDI=\;T_{c}-T_{a}^{∙}C \end{array}$$

where T_c_= canopy temperature at midday and T_a_ = air temperature at mid day.

A line quantum sensor was placed across the row 25 cm above the crop canopy to measure the incident radiation. The instrument was lowered down the canopy horizontally and placed above the soil surface to measure the radiation at the bottom. The reflected PAR was measured from the same position by simply inverting the sensor. The PAR use efficiency (PARUE) was estimated using Equation 5:

(5) $$\begin{array}{r} PARUE\left[gMJ^{-1}\right]=\;\frac{Drymatteraccumulation\;\left(gm^{-2}\right)}{AccumulatedabsorbedPAR\;\left(MJm^{-2}\right)} \end{array}$$

where accumulated absorbed PAR (APAR) was estimated using Equation 5.1.

(5.1) $$\begin{array}{r} \text{APAR}=\left[\text{PAR}\left(\text{o}\right)+\text{RPAR}\left(\text{s}\right)\right]-\left[\text{TPAR}+\text{RPAR}\left(\text{c}\right)\right] \end{array}$$

where PAR (o) = the portion of the incident PAR above the canopy.

RPAR (s) = reflected PAR from the surface under the lentil canopy (reflected PAR value at the bottom level of crop.

TPAR = transmitted portion of the PAR through the canopy to the soil surface (Incident PAR value at the bottom of the crop).

RPAR(c) = reflected PAR from the crop (reflected PAR value at the uppermost layer of the crop canopy).

### Measurement of Soil Moisture

Soil moisture measurement was carried out gravimetrically. Moisture was recorded from three depths, viz., 0–15, 15–30, and 30–45 cm. All the samples were dried in an oven at 105°C for 24–48 h, so that the moisture present in the soil samples may be lost. Thereafter, all dried soil samples again were weighed on an electrical balance, and the readings were noted. Actual moisture content in each soil sample was calculated using Equation 6.

(6) $$\begin{array}{r} \text{Soil moisture }\left(\text{\%}\right)=\frac{Fresh\;weight\;of\;soil\;\left(g\right)-Dry\;weight\;of\;soil\;\left(g\right)}{Dry\;weight\;of\;soil\;\left(g\right)}\times 100 \end{array}$$

From percent soil moisture, soil moisture on a depth basis was estimated using Equation 7.

(7) $$\begin{array}{r} \text{Soil moisture }\left(\text{cm}\right)=\text{Soil moisture content }\left(\text{\%}\right)\times {\text{A}}_{\text{i}}\times D \end{array}$$

where A_i_ = apparent specific gravity of soil (or bulk density of soil, dimensionless) and D = depth of soil (cm).

The profile water contribution (ΔS) from soil at various depths was computed from moisture content of the soil. It was determined from the difference in soil moisture content at sowing, and harvesting of lentil and was estimated using Equation (8).

(8) $$\begin{array}{r} \Delta S=\overset{60}{\underset{i=10}{\sum }}\frac{M_{s}-M_{h}}{100}\times A_{i}\times D_{i} \end{array}$$

where ΔS–profile water contribution (mm); M_s_-moisture content of the soil at sowing (%); M_h_-moisture content of the soil at harvest (%); A_i_-apparent specific gravity of soil of ith profile (orbulk density of soil, dimensionless) and Di–depth of soil (mm) of the ith profile.

### Statistical Analysis

Statistical analysis of the data was performed applying the analysis of variance (ANOVA) technique of split-plot design (Gomez and Gomez, 1984). When the data were similar during both years, pooled analysis was carried out and presented. presented.ANOVA was conducted, and least significant values were calculated (*p* ≤ 0.05). Tukey's *post-hoc* test was applied to compare differences between the mean values.

## Results

### Temperature and Rainfall Prevailed During Lentil Growth

Daily distribution of rainfall and temperature (max and min) varied during the growth of lentil for the two consecutive years (Figures 1A,B) under different sowing dates. The range of temperatures at the time of flowering to pod formation (55–75 days) of lentil during 2018–2019 and 2019–2020 seasons were 6–28.4 and 9–32.2°C for the November-sown and 7.8–30 and 8.9–32°C for the December sown crops. Figure 1 shows that the crop received 16.2 cm of rainfall at vegetative to the flowering period (35–70 DAS) in the 2018–2019 season. However, the crop grown in the year 2019–2020 did not receive any rainfall during its vegetative phase.

### Effect of Different Date of Sowing and Foliar Spray of Micronutrients on Soil Moisture Distribution

**Figure 2:**
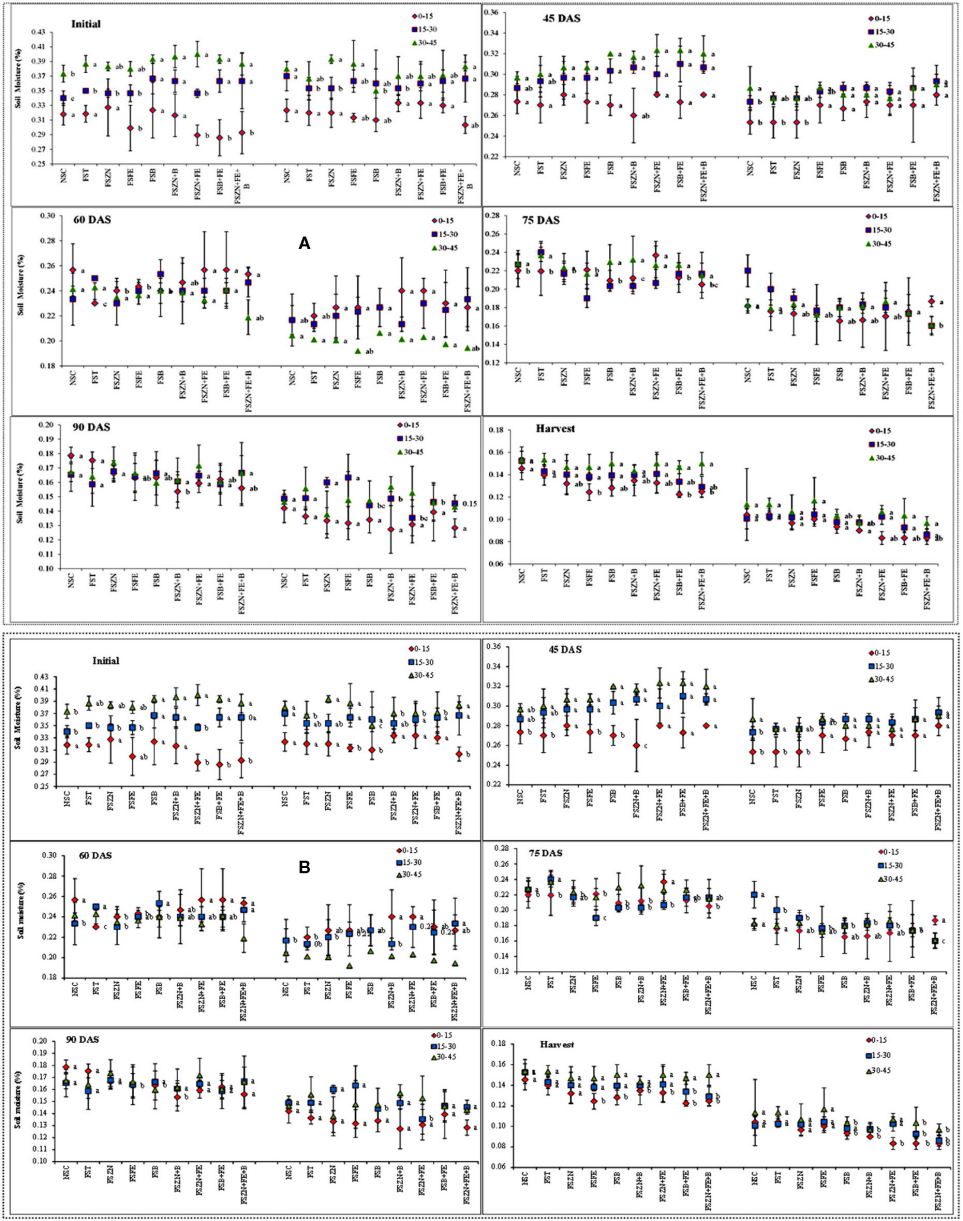
**(A)** Soil moisture content at different growth periods under different dates of sowing and foliar spray (data of 2018–2019 crop season) (error bars represent the standard error of mean, and different letters indicate significant differences between means); and **(B)** soil moisture content at different growth periods under different dates of sowing and foliar spray (data of 2019–2020 crop season) (error bars represent the standard error of mean, and different letters indicate significant differences between means). Treatments details are available in Table 1.

Temporal soil moisture distribution indicated a decreasing trend from sowing to harvesting (Figures 2A,B) at each depth in different tillage systems for two consecutive years. During sowing, the moisture content varied between 28 and 38% in the November sowing, and 30 and 38% in the December sowing across the depth (0–45 cm). It started depleting throughout the crop season. The foliar spray of micronutrients did not show any significant result across the treatments. However, the foliar spray of FS_B+FE_ and FS_B+FE+ZN_ reported slightly more soil moisture availability than the control in the later stages. In the year 2018–2019, the soil moisture content ranged from 35 to 37, 25 to 29, 20 to 24, 18 to 22, and 15 to 18% for sowing to vegetative (S–V), vegetative to flowering (V–F), flowering to pod development (F–P), pod development to maturity (P–M), and maturity to harvest (M–H) periods, respectively, for the November sown crop.

**Figure 3:**
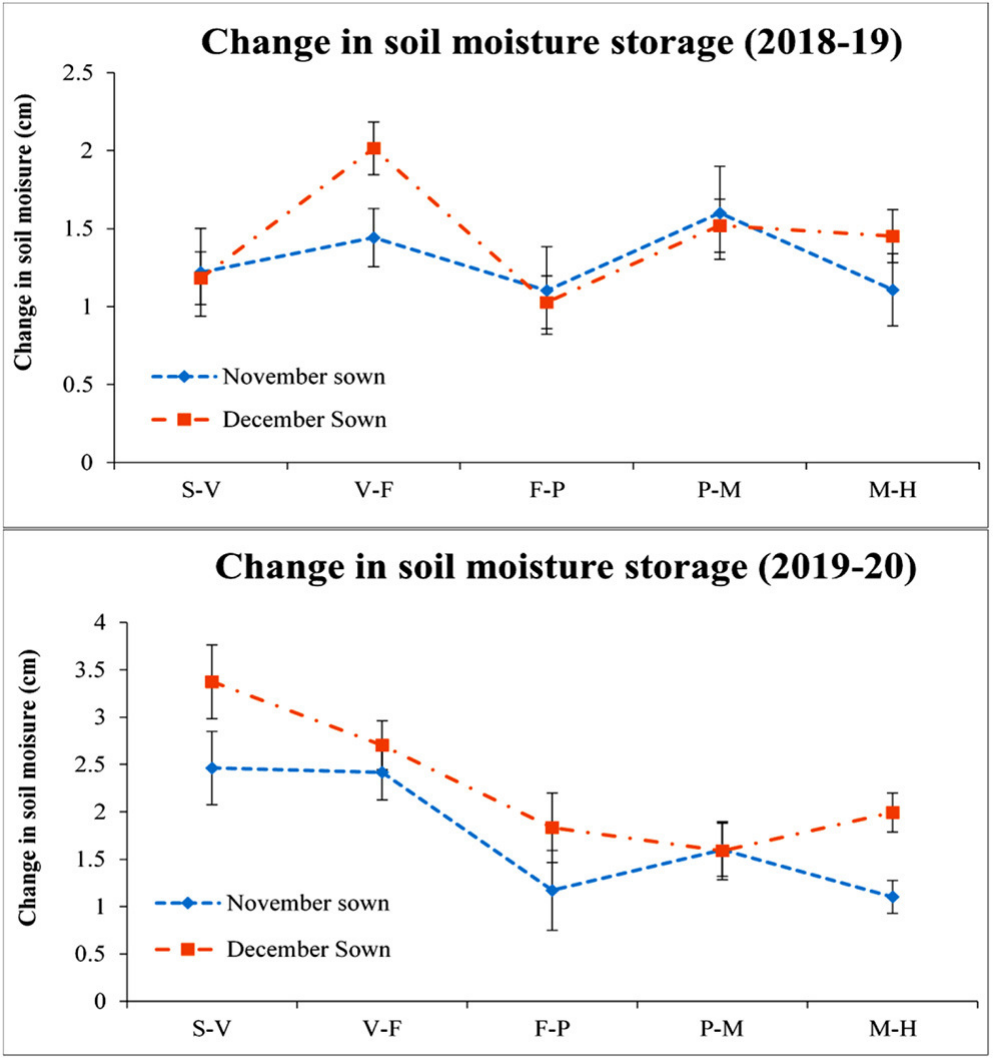
Change in soil moisture storage (0–45 cm soil profile) under different dates of sowing (data of 2 years (error bars represent the standard error of mean). S–V, sowing to vegetative; V–F, vegetative to flowering; F–P, flowering to pod development; P–M, pod development to maturity; and M–H, maturity to harvest.

The December sown crop was available with less soil moisture throughout the season when compared with the November sown one. It ranged from 30 to 33, 22 to 25, 16 to 19, 13 to 17, and 11 to 15% for the S–V, V–F, F–P, P–M, and maturity to harvest (M–H) periods. In the year 2019–2020, throughout the root zone (0–45 cm), the soil moisture content ranged from 33 to 36, 25 to 31, 18 to 25, 17 to 20, and 15 to 18% during the S–V, V–F, F–P, P–M, and M–H periods, respectively, for the November sown crop. Similarly, for the December sown crop, the soil moisture content varied from 30 to 32, 25 to 28, 25 to 28, 19 to 22, and 13 to 15% during S–V, V–F, F–P, P–M, and M–H periods, respectively. The change in soil moisture for both years also supports the data (Figure 3) December sown crop experienced a drastic reduction in the soil moisture content when compared to the November sown one, except for the initial soil moisture since the crop received a fair amount of rain (15.4 mm) just before its sowing.

### Effect of Dates of Sowing and Foliar Spray of Micronutrients on Physiological and Growth Traits of Lentil

**Figure 4:**
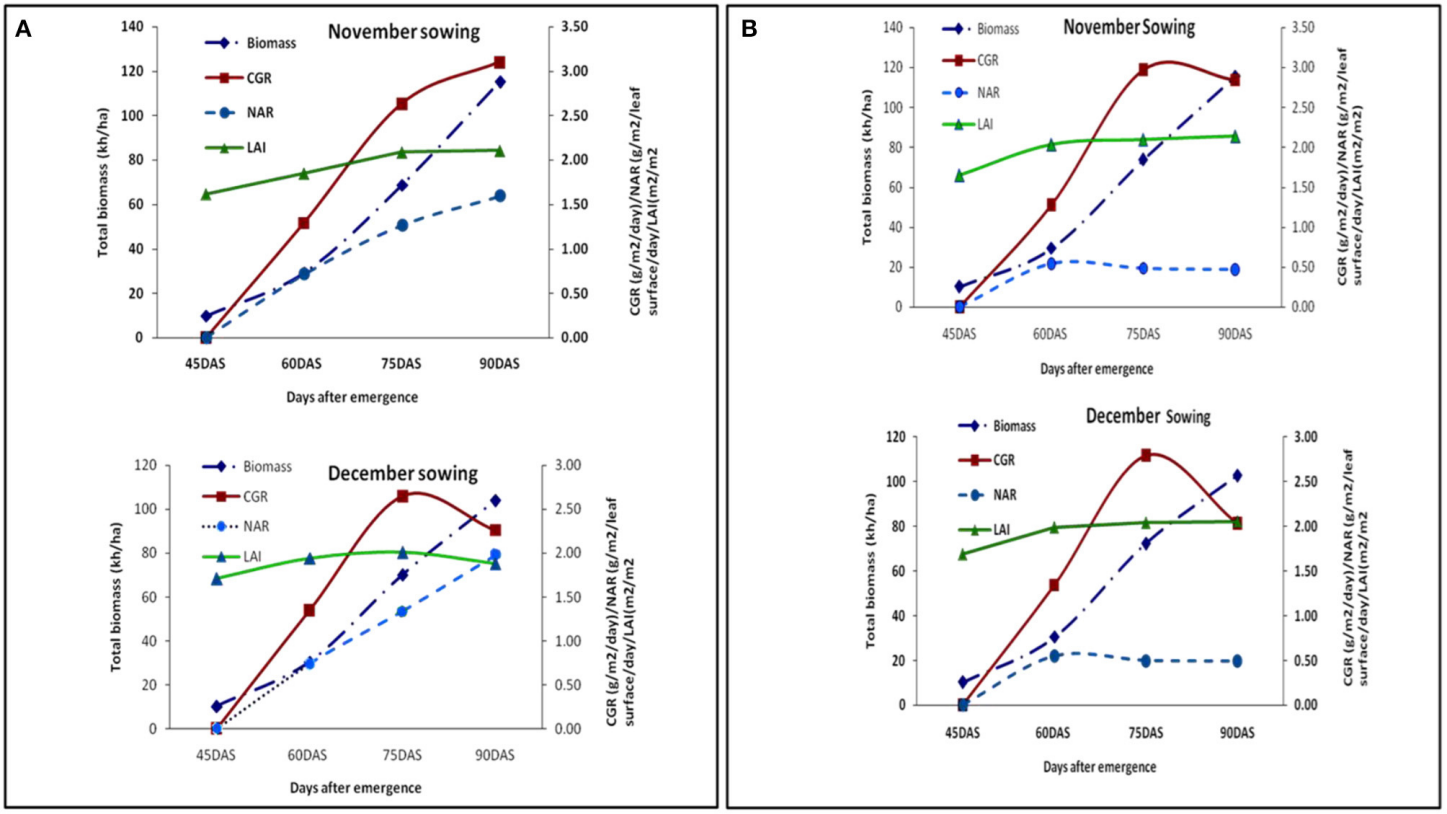
**(A)** Growth parameters at different growth periods under different dates of sowing (data of 2018–2019 crop seasons); **(B)** growth parameters at different growth periods under different dates of sowing (data of 2019–2020 crop seasons).

An increasing trend in LAI, CGR, and NAR from the early growing season was observed under both the dates of sowing in both the years. However, in the latter stages of the year 2018–2019, LAI and CGR decreased in the second date of sowing (Figure 4A). During the year 2018–2019, we observed heavy leaf fall in the later stages of the crop in the second sowing due to heavy wind prevalence during that period, thus decreasing the leaf area and crop growth rate.

LAI almost remained the same during the later stages, and CGR showed a decreasing trend (Figure 4B). Maximum LAI of 2.12 was observed for November and 1.97 was observed for December sown crops, respectively. Moisture stress (higher depletion rate) during pod development and maturity stages (Figure 2) reduced the LAI and CGR (Figures 4A,B). We observed only some significant changes in LAI with the foliar spray treatments and an increase in dry matter. The pooled data over 2 years of CGR, NAR, and biomass are presented in Table 3.

**Table 3:** Growth parameters of lentil influenced by date of sowing and foliar spray of micronutrients (pooled data of 2 years).

**Treatment**	**Net assimilation rate (g m^**−2**^ day^**−1**^)**	**Total dry matter (g/m^**2**^)**	**Crop growth rate (g m^**−2**^ day^**−1**^)**	**LAI**
November sowing	1.03 ± 0.05a	115.3 ± 0.91a	3.05 ± 0.08a	2.12 ± 0.04a
December Sowing	0.83 ± 0.09b	103.3 ± 2.35b	2.16 ± 0.16b	1.97 ± 0.02b
NS_C_	0.92 ± 0.05b	104.1 ± 1.02c	2.50 ± 0.09cd	1.96 ± 0.02b
FS_T_	0.99 ± 0.05ab	104.9 ± 0.62c	2.62 ± 0.07c	1.97 ± 0.02b
FS_ZN_	0.89 ± 0.02b	107.8 ± 1.42bc	2.44 ± 0.05d	1.98 ± 0.02b
FS_FE_	0.90 ± 0.10b	110.6 ± 3.31b	2.54 ± 0.05cd	2.03 ± 0.03b
FS_B_	0.93 ± 0.15b	109.9 ± 3.35b	2.56 ± 0.19cd	2.03 ± 0.02b
FS_ZN+B_	0.91 ± 0.04b	111.8 ± 0.70b	2.63 ± 0.25c	2.10 ± 0.07ab
FS_ZN+FE_	0.97 ± 0.06ab	111.8 ± 1.54b	2.73 ± 0.06b	2.06 ± 0.04b
FS_B+FE_	1.08 ± 0.06a	113.3 ± 1.23ab	3.05 ± 0.11a	2.12 ± 0.06a
FS_ZN+FE+B_	0.90 ± 0.09b	119.5 ± 1.27a	2.38 ± 0.12cd	2.16 ± 0.02a

*Values are means ±SEM. (n =3). Different letters indicate significant differences between means. Treatments details are available in Table 1*.

### Effect of Dates of Sowing and Foliar Spray of Micronutrients on Thermal Indices and Phenology

**Figure 5:**
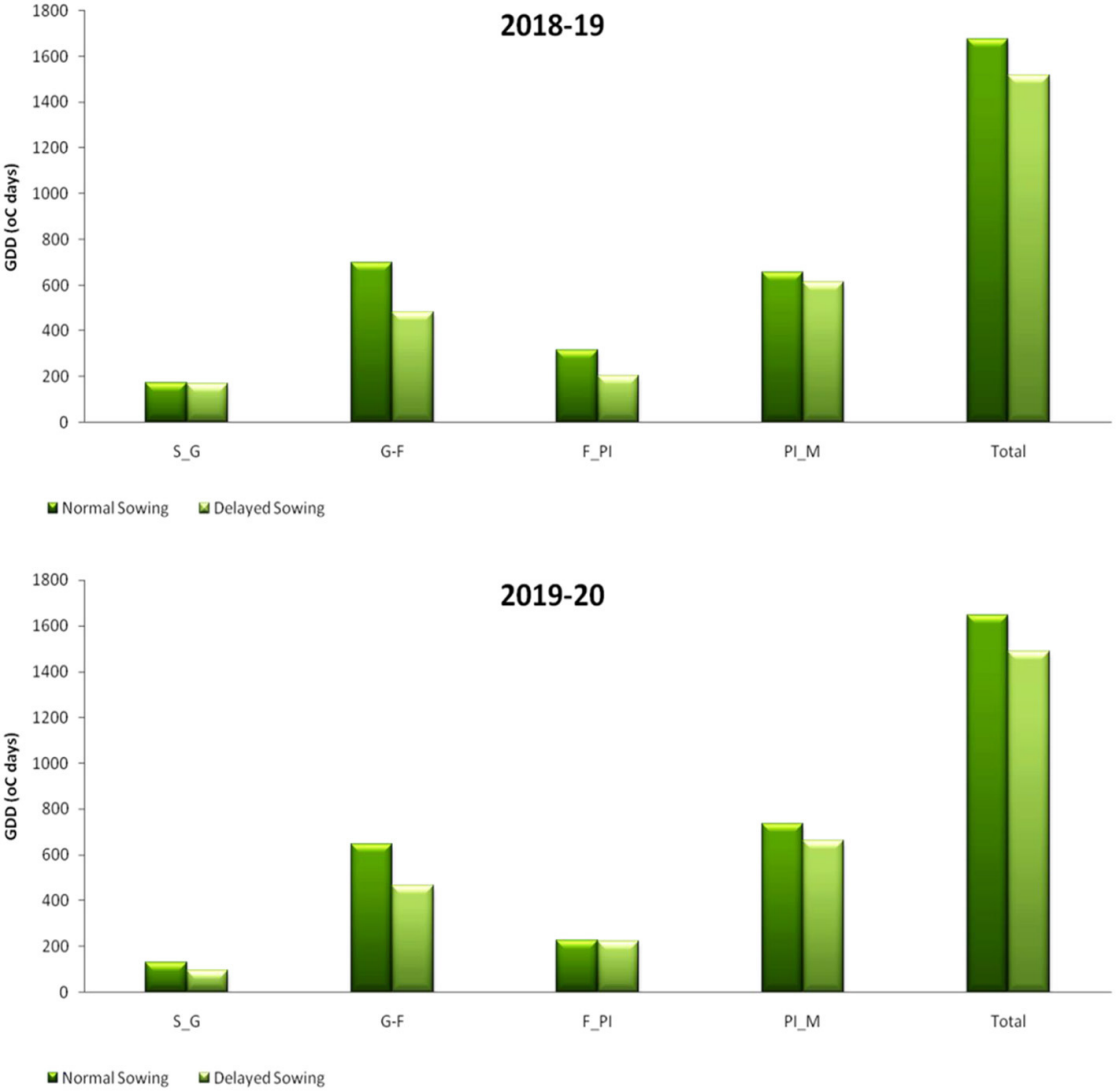
GDD at different growth period under different dates of sowing (data of 2018–2019 and 2019–2020 crop seasons). S–E, sowing to emergence; G–F, germination to flower initiation; F–PI, flower initiation to pod initiation; and PI–M, pod initiation to maturity.

The temperature in this region normally begins to rise from the end of February, reaches its maximum in May, and starts to decline from mid October, reaching a minimum of about 10°C by January. The variation in daily maximum and minimum temperature for 2 years during the crop season is given in Figure 1. During the first crop growing season, the total GDD accumulated was 1,676°C days for November sown crop and 1,514.9°C days for the December sown crop. The trend remained the same for the second crop season too. Mean GDD from sowing to emergence (S–E), germination to flower initiation (G–F), flower initiation to pod initiation (F–PI), pod initiation to maturity (PI–M), and total GDD accumulated for both the years are given in Figure 5.

Mean air temperature was higher for the December sown crop than the November sown one, which accelerated the phenological development of the later sown crop, especially during the flower initiation stage and early pod development stage. As lentil is an indeterminate crop, it keeps on producing flower as far as the source-sink relationship is maintained. SDD index is another thermal index that is largely used to estimate crop stress. Canopy temperature measurement is necessary to understand the plant-water status in a particular phenophase of a crop. The canopy temperature recorded at different stages of the crop is given in Table 4.

**Table 4:** Canopy temperature of lentil influenced by date of sowing and foliar spray of micronutrients (pooled data of 2 years).

**Treatment**	**Vegetative**	**Flowering**	**Podding**	**Maturity**
November sowing	22.8 ± 0.18b	24.4 ± 0.10b	25.8 ± 0.44b	28.5 ± 0.07b
December sowing	24.4 ± 0.22a	28.4 ± 0.24a	30.0 ± 0.24b	32.1 ± 0.26a
NS_C_	23.5 ± 0.12a	26.7 ± 0.05a	28.3 ± 0.10a	30.8 ± 0.05a
FS_T_	23.6 ± 0.23a	26.4 ± 0.15a	28.1 ± 0.27a	30.6 ± 0.06a
FS_ZN_	23.6 ± 0.15a	26.6 ± 0.16a	27.7 ± 0.31a	30.4 ± 0.47a
FS_FE_	23.6 ± 0.22a	26.5 ± 0.18a	27.8 ± 0.27a	30.4 ± 0.28a
FS_B_	23.6 ± 0.29a	26.5 ± 0.30a	27.9 ± 0.48a	30.3 ± 0.11ab
FS_ZN+B_	23.6 ± 0.20a	26.2 ± 0.25b	27.9 ± 0.39a	30.2 ± 0.05b
FS_ZN+FE_	23.5 ± 0.25a	26.3 ± 0.18b	28.2 ± 0.58a	30.0 ± 0.09b
FS_B+FE_	23.5 ± 0.21a	26.2 ± 0.15b	27.7 ± 0.44a	29.8 ± 0.16b
FS_ZN+FE+B_	23.5 ± 0.12a	26.2 ± 0.12b	27.5 ± 0.20a	29.9 ± 0.22b

*Values are means ± SEM (n = 3). Different letters indicate significant differences between means. Treatments details are available in Table 1*.

**Figure 6:**
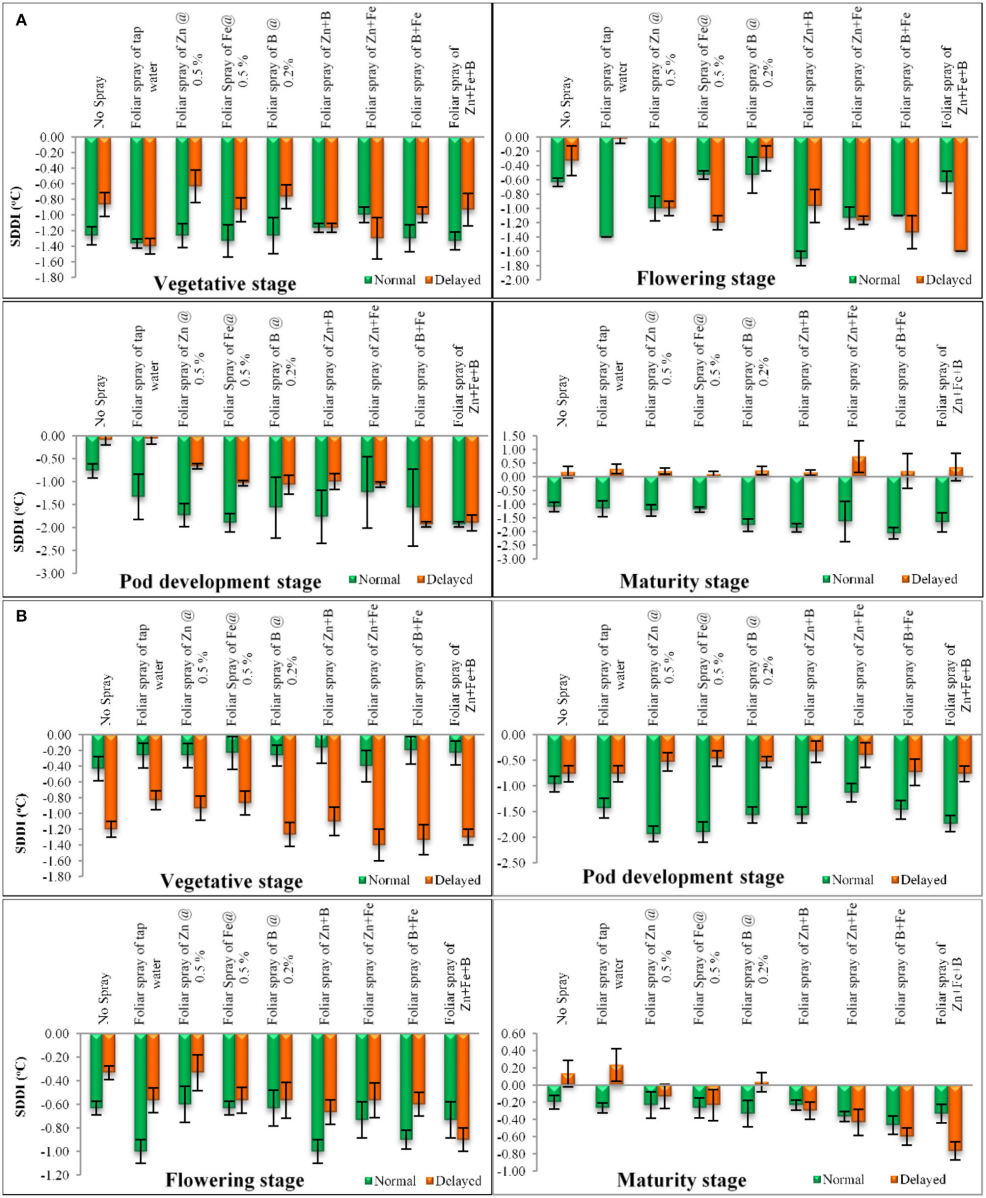
**(A)** SDD index at different growth period under different dates of sowing and foliar spray (data of 2018–2019 crop season) (error bars represent the standard error of mean, and different letters indicate significant differences between means.); **(B)** SDD index at different growth period under different dates of sowing and foliar spray (data of 2019–2020 crop seasons) (error bars represent the standard error of mean, and different letters indicate significant differences between means).

**Figure 7:**
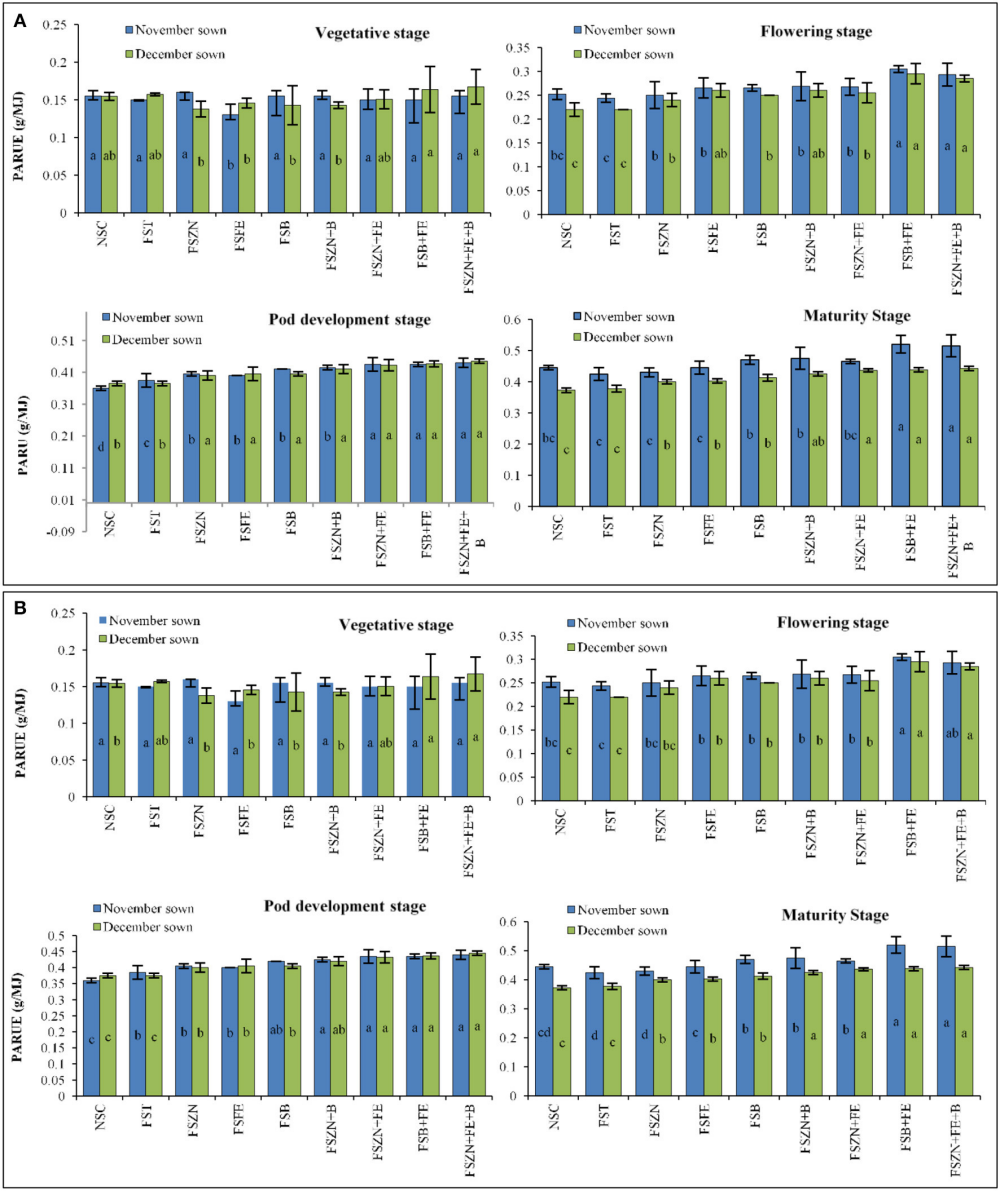
**(A)** PARUE at different growth periods under different dates of sowing and foliar spray (data of 2018–2019 crop season) (error bars represent the standard error of the mean, and different letters indicate significant differences between means.); and **(B)** SDD index at different growth periods under different dates of sowing and foliar spray (data of 2019–2020 crop season) (error bars represent the standard error of the mean, and different letters indicate significant differences between means). Treatments details are available in Table 1.

The normal sown crop recorded less canopy temperature when compared with the late sown crop. A variation of 3–5°C was observed at various growth stages. The increase in canopy temperature brought the crop under stress, as a result of which the late sown crop finished its life cycle almost 11 days earlier than the normal sown crop. However, this reduction in phenophase has a negative effect on crop yield and quality. The SDD index at various stages are presented in Figure 6. Lentil sown late recorded positive values at the later stage of crop growth (Figures 6A,B), indicating soil-moisture stress during this period. We have also observed low soil moisture as well as soil moisture storage at this stage (Figures 2, 3). The amount of intercepted PAR differed among sowing dates as well as treatments. The PARUE (g/MJ) among the treatments are given in Figures 7A,B.

**Figure 8:**
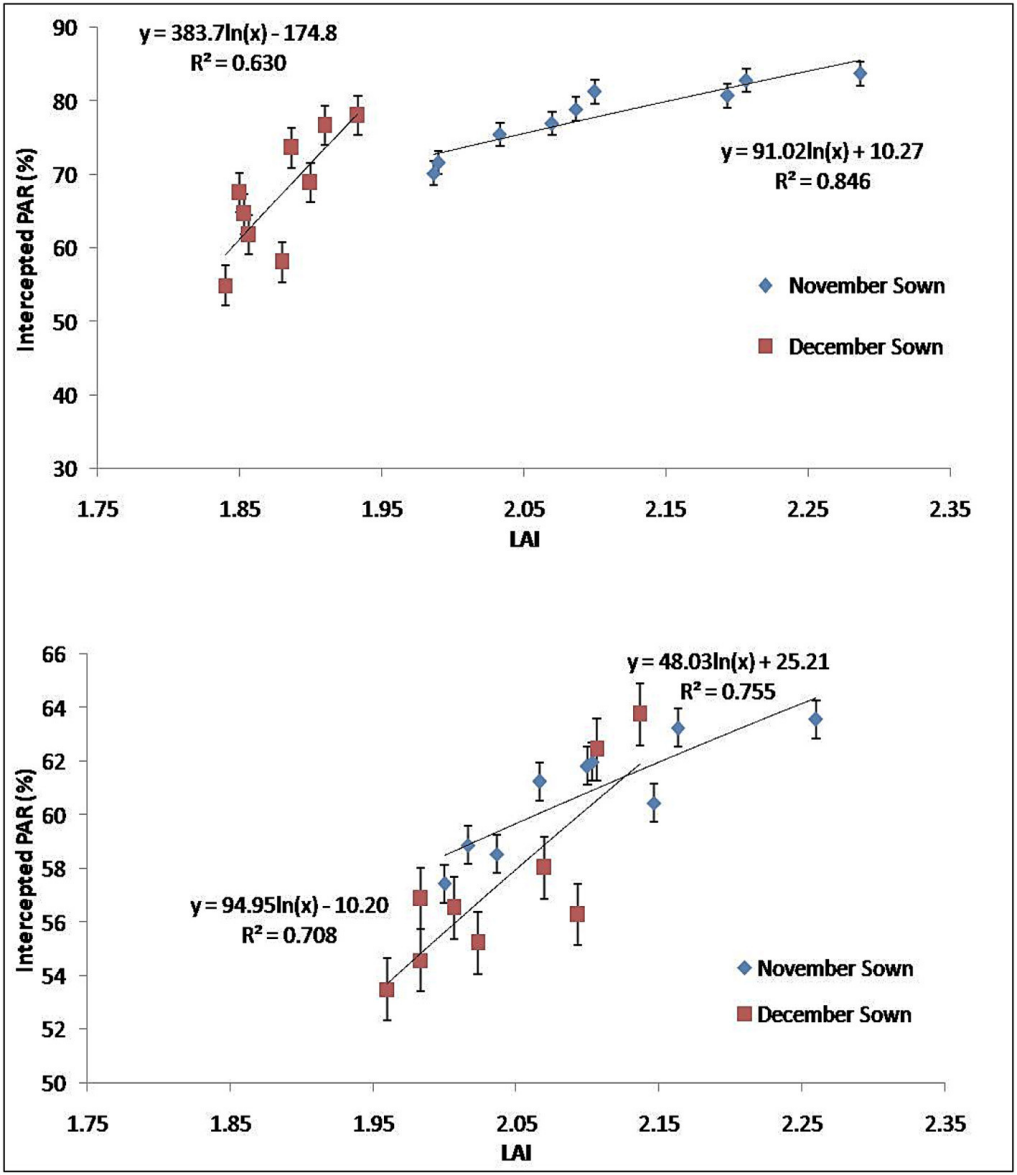
PARUE at the reproductive stage (75–90 DAS) under different dates of sowing and foliar spray (data of 2018–2019 and 2019–2020 crop seasons) (error bars represent the standard error of mean).

Marked differences were found in the amount of PAR intercepted between treatments up to the final harvest. In the year 2018–2019, PARUE ranged from 0.15 to 0.47 g/MJ in the November sown crop and 0.15– 0.43 g/MJ in the December sown crop (Figure 7A). In the year 2019–2020, PARUE varied from 0.15 to 0.47 g/MJ under November sown conditions and 0.15–0.41 g/MJ under December sown conditions (Figure 7B). There was a marked difference in the PARUE of the treatments. The foliar spray had a positive influence on the crop growth and crop canopy; and hence, the radiation absorption was also better. We observed an improvement of 14–18% more PARUE in crops that received foliar spray of FS_B+FE_ and FS_ZN+FE+B_ when compared with control during both the years at the reproductive stage. We also found that APAR was strongly and positively correlated with LAI at the reproductive stage of the crop in both years (Figure 8), clearly indicating a better canopy cover intercepts and absorbs more radiation.

The duration of the lentil crop was drastically reduced with a delay in sowing from November to December (Table 5). On average, the lentil sown on the first week of November took 113.5 days from sowing to maturity. However, the crop sown on the first week of December completed its life cycle in 102 days. Mean days for the crop sown on the normal date of sowing from sowing to emergence (E), Germination to flowering initiation (FI), Flowering to pod initiation (PI), and maturity (M) were 7.1, 45.8, 17.2, 45.3, and 113.5. On the other hand, the delayed date of sowing finished the stages of growth in 8.1, 39.1, 19.1, 35.9, and 102.1 days, respectively.

**Table 5:** Phenology of lentil influenced by date of sowing and foliar sprayof micronutrients (pooled data of 2 years).

**Treatment**	**S–E**	**G–F**	**F–PI**	**PI–M**	**LC: S–M**
November sowing	7.1 ± 0.00b	45.8 ± 0.62a	17.2 ± 0.36b	43.3 ± 0.67a	113.5 ± 0.91a
December sowing	8.1 ± 0.32a	39.1 ± 0.00b	19.1 ± 0.55a	35.9 ± 0.57b	102.1 ± 0.95b
NS_C_	7.8 ± 0.00a	41.8 ± 0.05a	18.2 ± 0.57a	38.6 ± 0.57b	106.3 ± 1.07a
FS_T_	7.6 ± 0.28a	42.3 ± 0.05a	17.7 ± 0.78b	39.1 ± 0.57b	106.8 ± 1.05a
FS_ZN_	7.4 ± 0.28a	42.1 ± 0.28a	18.1 ± 0.76a	39.4 ± 0.50b	107.2 ± 0.85b
FS_FE_	7.6 ± 0.00a	42.4 ± 0.86a	17.7 ± 0.28b	39.6 ± 0.28ab	107.3 ± 0.78b
FS_B_	7.7 ± 0.28a	42.5 ± 0.57a	17.8 ± 0.57b	40.0 ± 0.57a	108.0 ± 1.00a
FS_ZN+B_	7.7 ± 0.28a	42.7 ± 1.00a	18.3 ± 0.28a	39.6 ± 0.57ab	108.2 ± 1.07a
FS_ZN+FE_	7.6 ± 0.28a	42.8 ± 0.86a	18.4 ± 0.57a	39.6 ± 0.78ab	108.2 ± 1.26a
FS_B+FE_	7.5 ± 0.00a	42.9 ± 0.28a	18.5 ± 0.28a	40.3 ± 0.28a	109.3 ± 0.78a
FS_ZN+FE+B_	7.8 ± 0.00a	42.8 ± 0.57a	18.5 ± 0.00a	40.6 ± 0.29a	109.4 ± 0.05a

*S–E, sowing to emergence; G–F, germination to flowering; F–PI, flowering to pod initiation; PI–M, pod initiation to maturity; LC–S–M, life cycle sowing to maturity; Values are means ± SEM (n = 3). Different letters indicate significant differences between means. Treatments details are available in Table 1*.

### Yield of Lentil Is Influenced by Date of Sowing and Foliar Spray of Micronutrients

**Figure 9:**
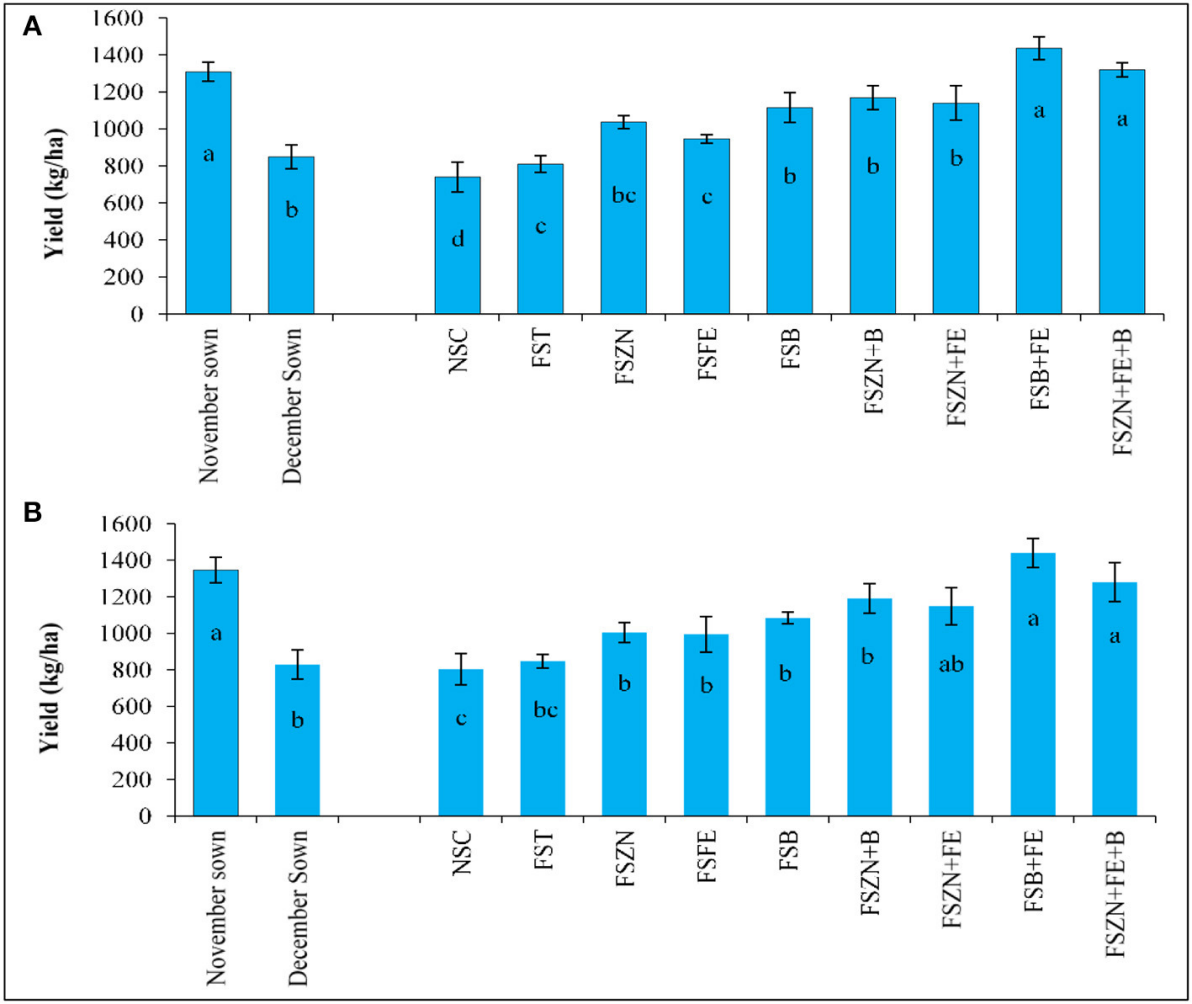
Yield for **(A)** 2018–2019; and **(B)** 2019–2020 of lentil is influenced by the date of sowing and foliar spray of micronutrients. Treatments details are available in Table 1.

With a maximum duration of the growth period, less soil moisture and temperature stress for the November sown crop led to higher grain yield (Figure 9). On an average, 1,309 and 1,347 kg ha^−1^ yields were obtained from the November sown crop, which were almost 33% more than those from the December sown crop. Among the micronutrient foliar spray treatments, FS_B+FE_ resulted in the highest yield in both the years (1,436 and 1,439 kg ha^−1^).

## Discussion

### Moisture Availability and Storage

According to Yadav et al. (2007), lentil needs low temperatures at the time of vegetative growth, while maturity requires warm temperatures; the best temperature for its optimum growth has been found to be 30/18°C (max/min), and above 32/20°C (max/min) during flowering and pod filling in lentil can drastically reduce seed yield and quality (Sita et al., 2018). The experiments revealed that the cooling periods were shorter, and that the heat periods were becoming longer under late sowing, further resulting in exposure of cool-season crops to heat stress and moisture stress, particularly in the reproductive stage. From Table 2, it is clear that during the reproductive stage (pod initiation to maturity), there was a difference of 2–3°C between the November and December sown crops in both the years. Moreover, there was an incidence of more than 32°C (Tmax) for 3–5 consecutive days. An increase in temperature beyond 32/20°C (Tmax/Tmin) is reported to severely affect the yield of the crop. This finding is in accordance with that reported earlier (Saxena, 2009; Malik et al., 2015). In both years, the crop was exposed to supra-optimal temperatures, especially when sown late. Since the crop is also raised without irrigation, along with heat stress, the crop also experienced severe moisture stress during the terminal stage, particularly when sown late. The severity increases when the crop faces both terminal heat and moisture stress together. Though late sowing of crop had lesser moisture availability, there was no significant effect of foliar spray except for FS_B+FE_ and FS_B+FE+ZN_ reporting slightly more soil moisture availability than the control at the later stages probably due to a better foliage cover.

### Physiological Parameters

Though the November sown crop is the appropriate time for sowing, the farmers in this region could not take up this time of sowing. Since in a larger situation a long duration rice crop (more than 150 days) is taken up, the sowing of lentil always gets delayed to the end of November or December. In agreement with the previous reports, the yield of any particular lentil variety is low when no alleviation measures are taken up. In this study, we found better growth with the application of foliar spray, especially FS_B+FE_. An increase in foliar growth of plants leading to more LAI and CGR, resulted in establishing a better canopy coverage, reducing the soil evaporation and thus higher soil moisture. According to Saxena (2009), the presence of abundant soil moisture greatly delays the maturity of lentil. Higher initial soil moisture in the soil profile under November sown lentil crop (after the harvest of rice), resulted in water availability for a longer period. The decrease in NAR and CGR may be due to low water uptake and higher transpiration rate under stress conditions (Talukdar, 2013). The rate of CGR to LAI indicates the NAR of the crop. An increasing or decreasing trend in LAI might promote the mutual shading of the leaves. Whenever the shading increases, it leads to a decrease in the average NAR of the leaves of lentil with an increase in the growth period (Sinclair and Muchow, 1999). The LAI of the crop gradually increased until early pod development, after which it started declining in the late sown crop because of a reduction in phenology due to stress. However, the normal sown crop maintained it until maturity. It was pointed out by Chaturvedi et al. (1980) that dropping of lower leaves did not contribute to photosynthesis but retained respiratory activity. Lower NAR at the later stages might have resulted in a lower photosynthetic rate. However, the respiratory activity would have remained high, leading to plant stress. In cluster bean, similar effects of soil moisture stress on NAR have been observed (Vyas et al., 2001).

### Thermal Indices and Phenology

Changes in maximum and minimum temperatures reduce the duration of different phenological stages and adversely affect growth processes in crops (Parya et al., 2010). Any temperature change will bring a change in canopy temperature, which will affect the growth and development of a crop. Higher canopy temperature and SDD index negatively affect dry matter production and yield (Chakravarti et al., 2010). An increase in canopy temperature results in an increased rate of transpiration as diffusive resistance gets increased (Baligar et al., 2012). The interesting finding during this experiment was that in the late sown crop, the treatment with foliar spray of FS_ZN+FE_, FS_B+FE_ and FS_ZN+FE+B_ recorded a negative SDD index value at this stage. This results from prominence the fact that micronutrients like zinc, iron, and born can ameliorate the increase in temperature to a better extent. Solar radiation is important for efficient photosynthesis. The absorption of photosynthetically active radiation depends on the canopy and duration of the crop. The higher interception of PAR in November sowing over December sowing was due to the longer duration. The November sown crop also has a higher canopy cover, which is clear from its LAI. Similar results were reported in lentil (Azam et al., 2002) and chickpea (Hussain et al., 1998). Improved PARUE is a clear indication of a high photosynthetic rate. All these factors might have led to improved productivity in crops that were sprayed with a foliar application of micronutrients.

The phenology of the crop reduced when the sowing was delayed. As the temperature started increasing and the crop started experiencing moisture and heat stress, the life cycle of the crop started reducing. A reduction in time to 50% flowering and maturity for the December sown lentil (cv. LG 308) was observed by Singh et al. (2005) and Sen et al. (2016). Though the differences between the stages are not standardized, the study found that G–F and PI–M influenced the life cycle of the lentil crop as a whole. The emergence of lentil seedlings was faster (7 days) in normally sown plots than in later sown dates, i.e., delayed (8 days), which may be due to better residual soil moisturization.

### Yield of Lentil

We could observe from the SDD index and canopy temperature results that the crop experienced stress during the later stage of growth (reproductive stage). It has been reported that the reproductive stage is more sensitive to moisture and heat stress. The reproductive stage of growth is more sensitive to drought than the vegetative stage, resulting in poor seed set and, hence, affecting the yield (Pushpavalli et al., 2014). We could, however, observe that when the crop is sown late, the application of foliar nutrients can help in ameliorating the adverse effect of heat and moisture stress and help in reducing the stress, thereby improving the yield (Roy et al., 2009). Since lentil was raised as a rain-fed crop, the change in soil moisture storage has a big role to play. The increase in temperature along with the soil moisture stress has resulted in differences in the length of the growth period and, hence, the yield of the crop is drastically affected (Tiwari and Vyas, 1994). Change in higher soil moisture storage in the November sown crop compared with the December sown crop from sowing to harvesting resulted in less residual soil moisture evaporation or less transpiration. Apart from that, foliar spray of micronutrients, especially FS_B+FE_ and FS_ZN+FE+B_ resulted in ameliorating the effect of both heat and moisture stress to an enhanced extent. This result is in close agreement with Tuti et al. (2012) and Visha Kumari et al. (2019) in humid tropical and sub-tropical climates. Early sowing has an advantage in enabling the long flowering to pod filling period (70–100 DAS) that may influence fruit setting and yield. Foliar spray, though not very evident, has some effect on keeping the crop to flower more as lentil is an indeterminate crop.

## Conclusions

The effect of sowing date and application of foliar spray of micronutrients implies an improvement in crop yield of lentil crop in Eastern India. This study found that the soil moisture storage experienced a continual reduction from sowing (relatively high storage) to harvest (much-reduced storage). However, it is highly influenced by the date of sowing and foliar spray (to some extent). Rapid depletion of soil moisture, along with an increase in air temperature in delayed sowing, increased soil moisture and plant heat stress, especially during the reproductive phase of the crop. The LAI, CGR, and NAR could be considered as important indices for the better expression of plant water stress. Canopy temperature, SDD index, phenology, and PARUE could be better indicators for heat stress. This study has concluded that appropriate time of sowing, along with foliar spray of micronutrients, could result in higher yield. Under delayed sowing conditions, foliar spray of boron at the rate of .2% and iron at the rate of .5% would help in reducing stress and could help farmers in sustaining better yield, especially during the rice-lentil cropping system.

## Data Availability Statement

The original contributions presented in the study are included in the article/supplementary material, further inquiries can be directed to the corresponding author/s.

## Author Contributions

VV, RN, and KS: conceptualization, validation, and investigation. VV and RN: methodology. VV: software and visualization. VV, AN, SB, and MC: formal analysis. RN and KS: resources and supervision. VV and AH: data curation. VV, RN, KS, AN, SB, and MC: writing—original draft preparation. ED, AA, MH, and AH: writing—review and editing and funding acquisition. RN: project administration. All authors have read and agreed to the published version of the manuscript.

## Conflict of Interest

The authors declare that the research was conducted in the absence of any commercial or financial relationships that could be construed as a potential conflict of interest.
